# Automated Synthesis of [^68^Ga]Ga-FAPI-46 on a Scintomics GRP Synthesizer

**DOI:** 10.3390/ph16081138

**Published:** 2023-08-11

**Authors:** Elisabeth Plhak, Christopher Pichler, Björn Dittmann-Schnabel, Edith Gößnitzer, Reingard M. Aigner, Susanne Stanzel, Herbert Kvaternik

**Affiliations:** 1 Division of Nuclear Medicine, Department of Radiology, Medical University of Graz, Auenbruggerplatz 9, A-8036 Graz, Austria; christopher.pichler@medunigraz.at (C.P.); bjoern.dittmann-schnabel@medunigraz.at (B.D.-S.); reingard.aigner@medunigraz.at (R.M.A.); susanne.stanzel@medunigraz.at (S.S.); herbert.kvaternik@medunigraz.at (H.K.); 2 Department of Pharmaceutical Chemistry, Institute of Pharmaceutical Sciences, University of Graz, Schubertstraße 1/EG/0122, A-8010 Graz, Austria; edith.goessnitzer@uni-graz.at

**Keywords:** [^68^Ga]Ga-FAPI-46, fibroblast activation protein inhibitor, automated synthesis

## Abstract

[^68^Ga]Ga-FAPI-46 is a radiolabelled fibroblast activation protein inhibitor that selectively binds to fibroblast activation protein (FAP), which is overexpressed by cancer-associated fibroblasts (CAFs) in the tumour microenvironment. In recent years, radiolabelled FAP inhibitors (FAPIs) are becoming increasingly important in cancer diagnostics and also for targeted radionuclide therapy. Because of the increasing demand for radiolabelled FAPIs, automating the synthesis of these compounds is of great interest. In this work, we present a newly programmed automatic synthesis process of [^68^Ga]Ga-FAPI-46 on a Scintomics GRP module using two Galli Ad generators as a radionuclide source. Dedicated cassettes for the labelling of ^68^Ga-peptides were used without any modifications. The generators were connected via a three-way valve to the module and eluted automatically over a strong cation exchange (SCX) cartridge by using the vacuum pump of the synthesis module, eliminating the need to transfer the eluates into a separate vial. After a reaction step in HEPES buffer, the compound was purified by solid-phase extraction (SPE) over a Sep-Pak Light C18 cartridge. The evaluation of 10 routine syntheses of [^68^Ga]Ga-FAPI-46 resulted in a radiochemical yield of 72.6 ± 4.9%. The radiochemical purity was 97.6 ± 0.3%, and the amount of free gallium-68 and colloid was <2%. The final product fulfilled the quality criteria, which were adapted from relevant monographs of the *European Pharmacopoeia* (Ph. Eur.). This work presents the successful preparation of multiple doses of [^68^Ga]Ga-FAPI-46 in a GMP-compliant automated process for clinical use.

## 1. Introduction

Fibroblast activation protein (FAP) is a membrane-bound peptidase, which is overexpressed on cancer-associated fibroblasts (CAFs) in the tumour microenvironment [1,2]. These cells play a key role in tumour progression and metastasis by the secretion of growth factors and cytokines promoting cell proliferation, mutagenesis and angiogenesis [3].

FAP is a protein of the dipeptidyl peptidase 4 family with an endopeptidase activity. It can cleave structural proteins of the extracellular matrix and alter the tumour microenvironment, promoting cell invasion and the formation of metastasis. Moreover, FAP is associated with tumour progression by supporting angiogenesis, immunosuppression, and drug resistance [4,5,6,7].

In addition to its expression in the tumour microenvironment, increased FAP expression has also been detected in inflammatory diseases such as rheumatoid arthritis and fibrotic diseases. FAP expression in healthy adult tissues is very low or absent. It only occurs in very particular cell types, like stromal cells or mesenchymal stem cells during wound healing or embryogenesis [2,7].

Due to the high selective expression of FAP in the tumour microenvironment and its involvement in key processes of tumour growth and survival, it has become an interesting molecular target for the development of cancer therapies and diagnostics in recent years [8,9].

The first radiolabelled fibroblast activation protein inhibitor (FAPI) was the boronic-acid-based compound [^125^I]I-MIP-1232, which was tested in vitro [9]. However, the selectivity and affinity of this compound to FAP was low compared to its selectivity and affinity for other dipeptidyl peptidases (DPPs) and prolyl oligopeptidases (PREPs) [8,9].

Jansen et al. discovered a new group of FAPIs based on an N-(4-quinolinoyl)-Gly-(2-cyanopyrrolidin) scaffold [10]. Nowadays, there is a variety of different quinoline-based radiolabelled FAPI ligands under clinical evaluation [8].

FAPI-04 is a DOTA-conjugated compound and was the first radiolabelled tracer applied to patients in a theranostic setting. Patient investigations have been carried out with ^68^Ga-, ^90^Y- and ^177^Lu- radiopharmaceuticals [11,12].

Another improvement of this compound is FAPI-46 with a modified DOTA linkage component. This compound has enhanced pharmacokinetic properties compared to FAPI-04. Its high specific uptake into tumour tissue and low absorption into other organs qualifies its use in theranostics [13,14]. FAPI-46 labelled with gallium-68 is already successfully being used in cancer diagnostics. Labelled with lutetium-177 and yttrium-90, it also showed promising results in targeted radioligand therapy [14].

Due to the increasing demand for gallium-68-labelled FAPIs, automation of the labelling process has become more and more important. 

Up to now, automated syntheses have been carried out on a Modular Lab Pharm Tracer (MLPT), a Modular Lab eazy (ML eazy) [15], a Trasis All in One (Trasis AiO) [16], a Trasis Eazy One, a Synthra and a Scintomics GallElut module [17]. 

At the Division of Nuclear Medicine in Graz, we have developed a new automated process for the synthesis of [^68^Ga]Ga-FAPI-46 using a Scintomics GRP module using dedicated single-use kits without any modification. Our developed programme sequence includes the simultaneous elution of two Galli Ad generators by using the vacuum pump of the synthesis module, the preconcentration of the eluates over a SCX column and the purification of the labelled compound over a Sep-Pak Light C18 column. This work presents a reliable synthesis procedure for the preparation of [^68^Ga]Ga-FAPI-46 according to the GMP and the current good radiopharmaceutical practice (cGRPP) standards of the EANM [18]. 

## 2. Results

### 2.1. Radiolabelling

The Scintomics GRP module was equipped with the dedicated valve benches. Before the reagents and the precursor were added, a “prepare-load” programme including a vacuum test was carried out. Furthermore, the Galli Ad generators were prepared for the elution by manually turning the rotary knob to the ON position for 10–15 s. The knob was moved to the OFF position again, and the automatic synthesis was initiated with the preconditioning of the Sep-Pak Light C18 cartridge (Waters, Milford, MA, USA) connected to valve 4 and 5 with ethanol and water. 

**Figure 1:**
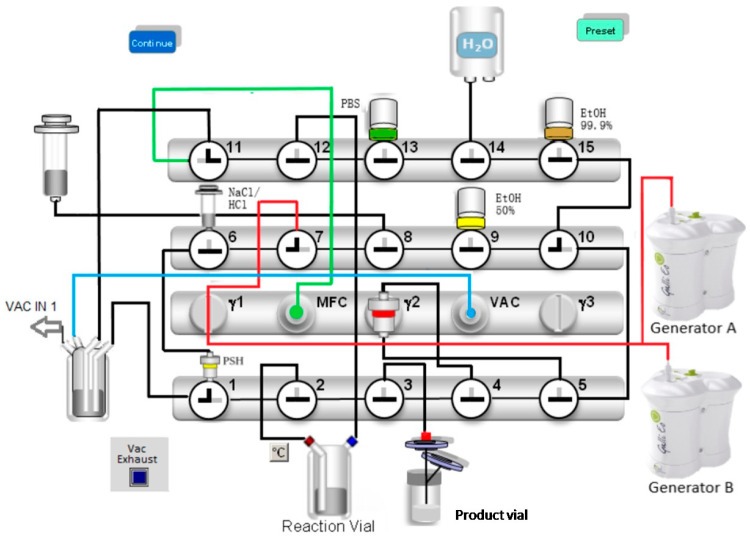
Configuration of the Scintomics GRP module for the synthesis of [^68^Ga]Ga-FAPI-46. The valves are numbered from 1 to 15. The figure shows the valve positions during the elution of the generators: valve 1: position 4, valve 7: position 4, valve 10: position 4, valve 11: position 2. All other valves remain closed (position 3).

In the next 60 s, a vacuum was built up over the valve benches 2 and 3 including the SCX cartridge, until the pressure decreased below 200 mbar. The transfer of the [^68^Ga]GaCl_3_ eluates was started by the opening of valve 7 (Figure 1). The vacuum suction transferred the eluates from the generators through the SCX cartridge, where gallium-68 was trapped. After 3 min, the transfer process was finished, and the vacuum was released by using nitrogen gas (N_2_). The synthesis continued by eluting [^68^Ga]GaCl_3_ from the SCX cartridge to the reaction vial with 5 M NaCl +150 µL 6 M HCl by creating a partial vacuum over valve 12 using the syringe pump. The radiolabelling of FAPI-46 was carried out at 125 °C in 6 min. The labelled compound was loaded on a Sep-Pak Light C18 column and purified with water through two washing steps. The product was eluted with 2 mL ethanol/water (1/1) over a sterile filter into the product vial. A portion of 15 mL PBS was used to rinse the delivery tubes to the product vial. The total synthesis time was 38 min.

Two Galli Ad generators were eluted simultaneously for the production of [^68^Ga]Ga-FAPI-46. The elution yield of the used Galli Ad generators was averaged to 60% in relation to the generator’s nominal activity. Based on this, a total starting activity of 1652 ± 238 MBq (n = 10) was calculated. Seven batches of [^68^Ga]Ga-FAPI-46 yielded a mean product activity of 863 ± 129 MBq. Based on the eluted total activity, the average decay-corrected radiochemical yield was 72.6 ± 4.9%. 

### 2.2. Quality Control 

The quality criteria of the final solution of [^68^Ga]Ga-FAPI-46 were adapted from the monographs 2482 [^68^Ga]Ga-DOTA-TOC injection and 3044 [^68^Ga]Ga-PSMA-11 injection of the *European Pharmacopoeia* [19,20]. 

The radionuclide identity was analysed by gamma spectrometry using a well-type scintillation detector. The characteristic decay energies of gallium-68 are at 511 keV and 1077 keV, according to the *European Pharmacopoeia* [19,20]. The energy line at 1022 keV (sum peak) and 1077 keV can be seen as a single peak in the spectrum of our NaI(Tl) scintillation detector. The half-life determination was carried out with a dose calibrator. 

The radiochemical identity of the product was tested using Radio-HPLC by comparing the retention time of the labelled compound (radio trace) with the retention time of the inactive (cold) compound [^nat^Ga]Ga-FAPI-46 (UV trace). Under the conditions mentioned in the Materials and Methods section, the retention time of [^68^Ga]Ga-FAPI-46 is expected to be about 8.5 min, which corresponds to the retention time of the standard. 

**Figure 2:**
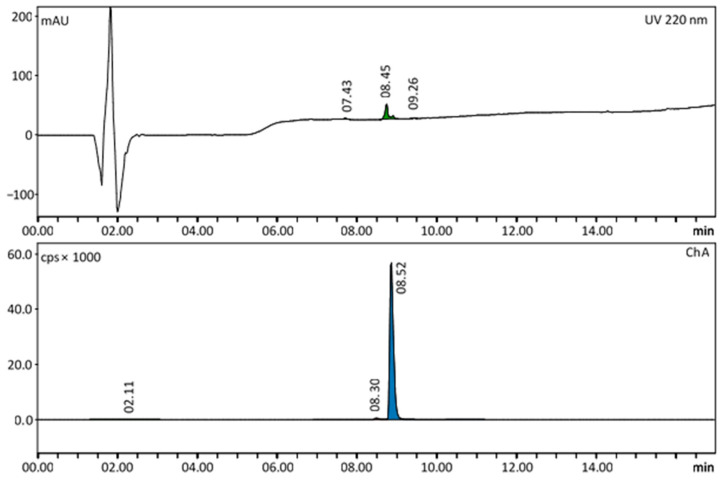
Representative Radio-HPLC chromatogram of a [^68^Ga]Ga-FAPI-46 product solution. In the radio trace, there is a visible impurity at 8.30 min (1.51%) below the principal peak of the labelled peptide (8.52 min, 98.2%). In the UV trace, the peaks at 8.45 min and 9.26 min are assigned to [^68^Ga]Ga-FAPI-46, cold FAPI-46 and metal impurities (3.29 µg/mL). The peak at 7.43 min was assigned as an unspecific impurity (0.21 µg/mL).

**Figure 3:**
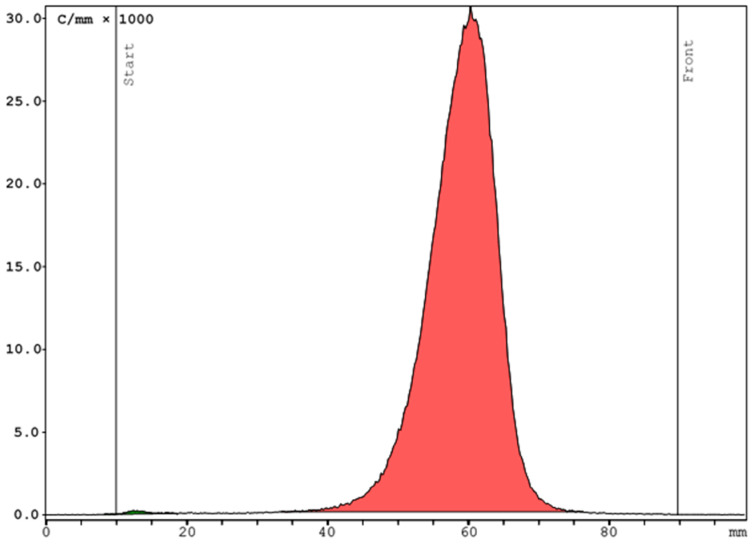
Representative Radio-TLC chromatogram of [^68^Ga]Ga-FAPI-46 using iTLC SG plates as a stationary phase and a 1:1 mixture of methanol and a 1 M ammonium acetate solution as a mobile phase. The radiolabelled compound [^68^Ga]Ga-FAPI-46 and [^68^Ga]GaCl_3_ (99.8%) moved to the front, while colloidal gallium-68 (0.2%) remained at the start.

The product met the specifications of radiochemical purity. The peak corresponding to [^68^Ga]Ga-FAPI-46 in the HPLC radio trace was 97.6 ± 0.3%, free gallium-68 amounted to ≤1%. The non-radioactive impurities were evaluated with the UV trace at a wavelength of 220 nm. A representative Radio-HPLC chromatogram of a product solution of [^68^Ga]Ga-FAPI-46 is shown in Figure 2. Colloidal gallium-68 was determined by Radio-TLC and constituted 0.2 ± 0.1%. A representative Radio-TLC chromatogram is shown in Figure 3. The amount of HEPES was tested after release and was ≤20 µg/mL. Bacterial endotoxins were under the limit of quantification of the used Limulus Amebocyte Lysate (LAL) assay (≤0.05 IU/mL). Other post-release tests included the radionuclide purity, the ethanol content and the sterility. The results of the quality control of 10 batches of [^68^Ga]Ga-FAPI-46 are summarized in Table 1. 

### 2.3. Stability Testing 

**Figure 4:**
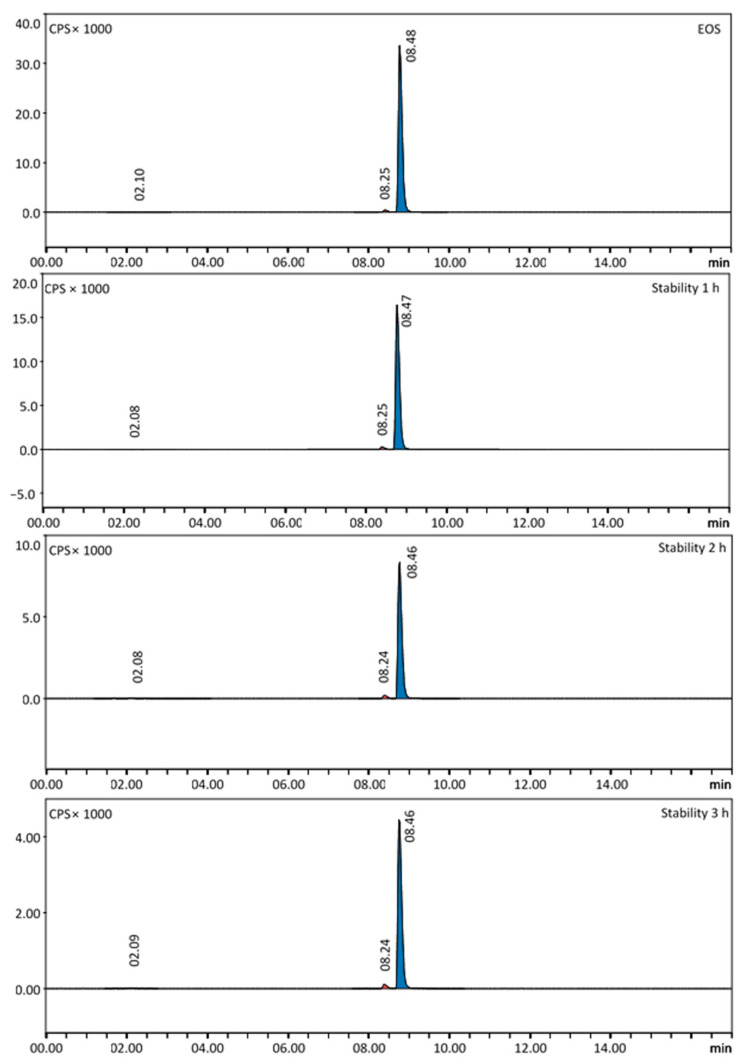
Radio-HPLC chromatograms of the stability testing of [^68^Ga]Ga-FAPI-46 over a period of 3 h. The final product had a radiochemical purity of 97.8% at EOS. After a time frame of 3 h, the radiochemical purity decreased to 96.1%, with a slight increase in free gallium-68 to 1%. The visible impurity with a retention time of 8.24 min increased from 1.8% (EOS) to 2.9% (3 h).

The stability of three batches of [^68^Ga]Ga-FAPI-46 was tested up to 3 h after preparation. With Radio-HPLC, we determined the amount of free gallium-68, which only minimally increased to a mean value of 1.4 ± 0.5% three hours after the end of synthesis (EOS). The amount of gallium-68 in colloidal form was measured with TLC and increased to an average of 1.0 ± 0.3% three hours after EOS. The mean radiochemical purity of the product was 95.3 ± 0.8% three hours after preparation. Representative Radio-HPLC chromatograms of the stability testing are shown in Figure 4. Detailed Radio-HPLC chromatograms for the presented chromatogram can be found in the Supplementary Materials (Figures S5–S8) as well as further Radio-HPLC chromatograms of the other two tested batches (Figures S1–S4 and S9–S12). The stability data of [^68^Ga]Ga-FAPI-46 are presented in Table 2. 

## 3. Discussion

In recent years, there have been some approaches to automate the synthesis of [^68^Ga]Ga-FAPI-46 using different synthesis modules in combination with various commercially available generators. 

The first automated preparation of [^68^Ga]Ga-FAPI-46 was published by Spreckelmeyer et al. In that work, a Modular Lab Pharm Tracer (MLPT) and a Modular Lab eazy module (ML eazy) were used in combination with ^68^Ge/^68^Ga generators from Eckert & Ziegler (EZAG). The generator eluate [^68^Ga]GaCl_3_ was purified and preconcentrated over an SCX column. The labelling was performed in an acetate/ascorbate buffer. By the measurement of radioactivity distribution for the MLPT module, 96% of the radioactivity was found in the productvial. Preparations up to 1700 MBq were reported, depending on the number of generators in use [15]. The preconcentration step allows the sequential elution of more than one generator using a motor syringe and collects all the [^68^Ga]GaCl_3_ onto the SCX for further processing [21]. 

Three different synthesis modules (a cassette-based Trasis Eazy One and a Synthra module in combination with EZAG generator and a valve and tubing Scintomics GallElut module in combination with an iThemba Generator) were used by Alfteimi et al. to carry out the synthesis of [^68^Ga]Ga-FAPI-46. This group did not use SCX purification and reported a radiochemical yield of approximately 93% with all three modules [17]. Naturally, a higher yield can be expected when the generator eluate is directly processed. In our experience preparing ^68^Ga-radiopharmaceuticals using Galli Ad and iThemba generators, we cannot recommend omitting the SCX purification. The possibility of a ^68^Ge-breakthrough or other metal impurities exists, especially when using non-pharmaceutical-grade generators. 

In contrast, the construction of the Galli Ad generator is completely different from that of the EZAG and iThemba. While the EZAG and iThemba generators need an external bag for the diluted HCl, the eluent of the Galli Ad generator is implemented as a factory-filled, internal tank. The elution of the [^68^Ga]GaCl_3_ will be performed using an evacuated vial. 

Da Pieve et al. reported the use of a [^68^Ga]GaCl_3_ eluate from a Galli Ad generator in the synthesis of [^68^Ga]Ga-FAPI-46. The eluate was obtained in an evacuated vial and further diluted with 1 mL of 0.1 M HCl before starting the automated synthesis. The “delivery vial” containing the diluted eluate was manually connected to the Trasis AiO synthesis module before the [^68^Ga]GaCl_3_ was collected onto an SCX cartridge and labelled in the acetate/ascorbate buffer [16].

To reduce the radiation dose of the operators, we developed an automated process in which the Galli Ad generators are directly connected to the apparatus for the preparation of ^68^Ga-peptides [22]. In this work, we present an optimized configuration of a Scintomics GRP module in combination with two Galli Ads to prepare [^68^Ga]Ga-FAPI-46. Our goal was the use of the commercially available GMP-grade kits for the labelling of ^68^Ga-peptides without any modification. The essential part is that the Scintomics GRP module system can create a vacuum of <200 mbar to achieve the complete elution of the Galli Ad generators. The vacuum is built up via the waste bottle by the internal pump of the module. A comparison of our work to the process of Da Pieve et al. [16] can be seen in Table 3. 

Before starting the Galli Ad elution, the vacuum test is advantageous to ensure that the system is airtight and the connections are properly fixed, to ensure the activity transfer into the module. The vacuum test starts by blowing both waste tubes with gas to make sure that any residual fluids within the tubing are removed. Afterwards, the valves to the SCX cartridge are opened and the vacuum pump is switched on. The operator monitors the decrease in pressure in the system via the SCC Scintomics software. If the pressure drops below 200 mbar, the pressure test is terminated by pressing the “Continue” button, and the system is vented. Subsequently, all valves are moved to the closed position. If a negative pressure < 200 mbar is not reached, the synthesis module is not ready for operation. In this case, all connections, valve positions and the functionality of the vacuum pump must be checked. If the vacuum test is successful, the assembly of the synthesis module can be continued by connecting the reagents, the precursor and the radionuclide generators.

Then, the Galli Ads are initialized manually according to the summary of product characteristics (SPC) by turning the rotary knob of the generator to the ON position for at least 10 s [23]. After turning the knob to the OFF position, the automated process is ready to start. A detailed description of the main process steps is presented in Table 4. 

The Galli Ad generators do not deliver 100% of the nominal activity. According to the SPC, the eluate from one generator has a fixed volume of 1.1 mL and contains over 55% of the total starting activity [23]. The measurements of six eluates from the used ^68^Ga-generators resulted in a radiochemical yield of 60.7 ± 1.5%. Therefore, an approximate initial activity of 60% of the nominal activity was used to calculate the radiochemical yield of [^68^Ga]Ga-FAPI-46.

The addition of ascorbic acid to the final solution is recommended in the literature to extend the shelf life of the final product [16,17]. We decided not to use any additives and calculated that the stability of the product was 95.3 ± 0.8% in a time frame of 3 h after EOS. However, we observed a slight increase in unbound gallium-68. If no stabilizer is added, the product should be administered within two hours after EOS to ensure a radiochemical purity of ≥95%.

## 4. Materials and Methods

### 4.1. Radiolabelling

The radiolabelling was carried out on a Scintomics GRP module (Fürstenfeldbruck, Germany). The “prepare-load vacuum test” sequence and the labelling sequence were programmed with the Scintomics developer software (SCC). The synthesis module was equipped with disposable cassettes for the labelling of ^68^Ga-peptides (SC-01-H) and dedicated reagents (SC-01) purchased from ABX (Radeberg, Germany). The 20 mL product vial (ABX, Radeberg, Germany) was equipped with a 0.2 µm Millex^®^ hydrophobic ventilation filter (Merck, Darmstadt, Germany) and a 0.22 µm Cathivex^®^-GV low-protein-binding sterile filter (Merck Millipore, Cork, Ireland). The product vial was connected to the product line starting from valve 3. 

Additional reagents for the synthesis like dimethyl sulfoxide (DMSO), hydrochloric acid (30%, ultrapure) and ultrapure water were purchased from Merck (Darmstadt, Germany). 

HCl (6M) was prepared by diluting 6.2 mL of 30% HCl with 3.8 mL of ultrapure water. 

The GMP-grade precursor (50 µg in a 5 mL brown glass vial) was obtained from SOFIE (Dulles, VA, USA). It was dissolved with 100 µL DMSO and diluted with 3.2 mL HEPES buffer. The justifications for the use of DMSO in the reaction solution can be found in Appendix A. Subsequently, the solution was transferred into the reactor vial with a pipette. HCl (6 M, 100 µL) was added for pH adjustment. Then, the reactor vial was connected with two tubing lines to valve 2 (main port) and valve 12 (ventilation port). 

The 5 mL glass vial containing 1.7 mL 5 M NaCl solution was opened, and 150 µL of 6M HCl was added. The solution was mixed and aspirated into a 3 mL syringe, which was mounted on the cassette (valve 6). 

Ethanol (99.9%, 5 mL, valve 15), ethanol/water (1/1, 2.0 mL, valve 9) and a 20 mL vial with phosphate-buffered saline (PBS, 20 mL, valve 13) were mounted to the cassette. The 250 mL water bag (water for injection, Braun, Melsungen, Germany) was connected with a spike and a tubing line to valve 14. 

^68^Ge/^68^Ga generators (Galli Ad) with a total activity of 1850 MBq at calibration date were purchased from IRE EliT (Fleurus, Belgium). The generators were equipped with the supplied polyethylene tubes (LectroCath) and a male–male Luer-lock connector (male-2-male LL) from Vygon (Ecouen, France). The tubes were connected to a three-way valve (Vycliccolor, Vygon, Ecouen, France) and connected with another polyethylene tube to the synthesis module (valve 7). The calibration dates of the generators were 3 months apart at the time of elution. 

### 4.2. Radionuclide Identity and Half-Life Determination

For gamma spectrometry, we used a well-type NaI(Tl) scintillation detector (Scionix, Bunnik, The Netherlands) connected to an ORTEC digiBASE with an integrated bias supply. Spectrum analysis was performed with the MAESTRO-32 MCA Emulation software (ORTEC/AMETEK, Oak Ridge, TN, USA). 

The determination of the approximate half-life was carried out with a dose calibrator ISOMED 2010 (NUVIA Instruments, Dresden, Germany).

### 4.3. Radiochemical Purity Testing by Radio-HPLC 

Radio-HPLC was performed on an Agilent 1260 series (Waldbronn, Germany) equipped with a DAD UV detector and a GABI Star radiometric detector (Raytest, Straubenhardt, Germany).

An ACE^®^3 C18 column (150 × 3.0 mm, Advanced Chromatography Technologies, Aberdeen, UK) was eluted by gradient elution (0.42 mL/min) with water/trifluoroacetic acid 0.1% (solvent A) and acetonitrile/trifluoroacetic acid 0.1% (solvent B): Start 5% B; 1–14 min 50% B, 16–17 min 5% B. The reference standard [^nat^Ga]Ga-FAPI-46 for quality control was kindly provided free of cost by SOFIE (Dulles, VA, USA). 

### 4.4. Radiochemical Purity Testing by Radio-TLC 

Radio-TLC was carried out by using iTLC-SG plates obtained from Agilent (Waldbronn, Germany) in combination with the standard solvent for the quality control for gallium-68-labelled peptides consisting of a 1:1 solution of methanol and 1 M ammonium acetate [19,20]. Measurements were carried out by using a MiniGITA Dual-Head Radio-TLC scanner (Elysia-Raytest, Straubenhart, Germany). The radiolabelled compound [^68^Ga]Ga-FAPI-46, as well as [^68^Ga]GaCl_3_, moved to the front, while gallium-68 in colloidal form remained at the start.

### 4.5. Evaluation of the pH Value 

The pH value of the product solutions was determined by using PEHANON pH indicator strips (Macherey-Nagel, Düren, Germany).

### 4.6. Post-Release Tests 

For the evaluation of the HEPES content, a validated HPLC method, modified from Antunes et al. [24], was used. A 150 × 4.6 mm XBridge C18 5 µm column (Waters, Milford, MA, USA) was eluted with a 20 mM solution of ammonium formate (pH 8) with a flow rate of 0.7 mL/min. The HEPES reference solution (40 µg/mL), the SST (system suitability test) standard with 2,5-dihydroxybenzoic acid and the samples were determined at UV λ = 195 nm.

For bacterial endotoxin testing, we used a quantitative kinetic chromogenic Limulus Amebocyte Lysate assay (Kinetic-QCL test kit, Lonza, Walkersville, MD, USA), apyrogenic 96-well microplates (Corning, NY, USA) and sterile and PCR-clean pipette tips purchased from Eppendorf. The measurements were performed at UV λ = 405 nm with a FLUOstar Omega microplate reader (BMG Labtech, Ortenberg, Germany) [25].

Accredited laboratory sites tested the ethanol content and sterility according to the *European Pharmacopoeia*.

## 5. Conclusions

Since [^68^Ga]Ga-FAPI-46 is a promising tracer for cancer diagnosis, there was a need for an automatic synthesis method in our clinic. An improved synthesis procedure using two Galli Ad generators was successfully applied for the preparation of [^68^Ga]Ga-FAPI-46. There were no changes in the configuration of the labelling cassette. Additionally, it was also not necessary to change the composition of the reagents or to use additional reagents. In summary, the presented synthesis of [^68^Ga]Ga-FAPI-46 is a reliable, GMP-compliant process, which can be implemented in everyday clinical practice.

## Figures and Tables

**Table 1:** Results of the quality control of 10 batches of [^68^Ga]Ga-FAPI-46.

Quality Control	Method	Criteria	Result (n = 10)
**Appearance**	visual inspection	clear and colourless	conforms
**pH value**	pH indicator strips	4–8	7.0
**Radioactivity concentration**	dose calibrator		50.8 ± 7.6 MBq/mL
**Radionuclide identity**	gamma spectrometry	511 keV, 1077 keV	conforms
**Approximate half-life**	dose calibrator	62–74 min	conforms
**Identity of [^68^Ga]Ga-FAPI-46**	Radio-HPLC	Retention time compared with cold reference	8.5 ± 0.04 min
**Colloidal gallium-68**	Radio-TLC	≤3.0%	0.2 ± 0.1%
**Free [^68^Ga]GaCl_3_**	Radio-HPLC	≤2.0%	0.6 ± 0.2%
**Radiochemical purity of [^68^Ga]Ga-FAPI-46**	Radio-HPLC	≥95.0%	97.6 ± 0.3%
**[^68^Ga]Ga-FAPI-46, FAPI-46 and related substances**	HPLC	≤5 µg/mL	4.1 ± 0.4 µg/mL
**Unspecific impurities**	HPLC	≤5 µg/mL	0.3 ± 0.1 µg/mL
**Ethanol content**	gas chromatography	≤10.0% (*v*/*v*)	≤6%
**HEPES content**	HPLC	≤500 µg/V	≤200 µg/V
**Bacterial endotoxins**	LAL test	≤175 IU/V	≤0.05 IU/mL (detection limit)
**Sterility**	Ph. Eur.	sterile	conforms

**Table 2:** Stability data of [^68^Ga]Ga-FAPI-46 product solutions (n = 3) up to 3 h after preparation.

Stability Testing	Acceptance Criteria	EOS	1 h	2 h	3 h
**[^68^Ga]Ga-FAPI-46 (HPLC) [%]**	≥95%	97.4 ± 0.4	96.2 ± 0.6	95.7 ± 0.7	95.3 ± 0.8
**[^68^Ga]GaCl_3_ (HPLC) [%]**	≤2%	0.6 ± 0.2	1.1 ± 0.3	1.2 ± 0.2	1.4 ± 0.5
**Gallium-68 in colloidal form (TLC) [%]**	≤3%	0.4 ± 0.2	0.7 ± 0.2	0.8 ± 0.2	1.0 ± 0.3

**Table 3:** Comparison between the described method of labelling [^68^Ga]Ga-FAPI-46 and the method of Da Pieve et al.

	This Work	Da Pieve et al. [16]
**Synthesis Module**	Scintomics GRP 3 V	Trasis AiO
**Radionuclide source**	Galli Ad Direct eluation	Galli Ad Delivery vial
**Purification of generator eluate**	SCX	SCX
**Labelling buffer**	HEPES 1.5 M	Acetate 0.2 M/ 1 mg sodium ascorbate
**Labelling temperature/time**	125 °C/6 min	95 °C/10 min
**Purification of the Product**	Sep-Pak C18	Oasis HLB + Sep-Pak QMA
**RCY d.c. (%)**	72.6 ± 4.9	66.0 ± 7.6

**Table 4:** Summary of process steps of the automated synthesis of [^68^Ga]Ga-FAPI-46.

**1.**	Preconditioning of the Sep-Pak Light C18 cartridge with 5 mL ethanol (99.9%) and 19 mL water.
**2.**	Drying of valve benches and the Sep-Pak Light C18 cartridge with nitrogen gas (N_2_).
**3.**	Preparing the elution of the generators by building up the vacuum until the pressure drops below 200 mbar.
**4.**	Simultaneous automatic elution of the Galli Ad generators over the PSH^+^ cartridge.
**5.**	Lifting the negative pressure by gently filling with N_2_.
**6.**	Eluting the gallium-68 from the PSH^+^ cartridge to the reactor vial with 1.5 mL 5 M NaCl (5 M)/150 µL HCl (6 M) using the syringe pump.
**7.**	Labelling at 125 °C/6 min in HEPES buffer (1.5 M).
**8.**	Transfer of the reaction mixture from the reaction vial onto the Sep-Pak Light C18 cartridge.
**9.**	Washing of Sep-Pak Light C18 cartridge with 14 mL water followed by a drying step with N_2_.
**10.**	Washing of the reaction vial with 10 mL water and drying with N_2_.
**11.**	Washing of Sep-Pak Light C18 Cartridge with 10 mL water followed by drying with N_2_.
**12.**	Elution of [^68^Ga]Ga-FAPI-46 to the product vial with 2 mL ethanol/water (1/1).
**13.**	Dilution of the product with 15 mL PBS.
**14.**	Washing of the valve benches with 10 mL PBS buffer and 10 mL water followed by a drying step with N_2_.
**15.**	Closing all valves—end of synthesis (EOS).

## Data Availability

Data are contained within this article and in the Supplementary Materials.

## Supplementary Materials

The following supporting information can be downloaded at https://www.mdpi.com/article/10.3390/ph16081138/s1: Figures S1–S4: Stability study of [^68^Ga]Ga-FAPI-46 batch 1, Figures S5–S8: Stability study of [^68^Ga]Ga-FAPI-46 batch 2, Figures S9–S12: Stability testing of [^68^Ga]Ga-FAPI-46 batch 3.

## Appendix A

During our pre-tests, we observed a better solubility of FAPI-46 in the presence of DMSO. The reaction mixture contains 100 µL of DMSO, which equals 110 mg based on a density of 1.1 g/cm^3^. As a water-soluble component, the DMSO is removed by the Sep-Pak Light C18 cartridge during the synthesis. According to monograph 04/2022:50400 “Residual Solvents” of the *European Pharmacopoeia,* DMSO is classified as a Class 3 solvent (less toxic and of lower risk to human health). Class 3 solvents have a PDE (Permissible Daily Exposure) of 50 mg or more per day [26]. According to our measurements (each batch) of the concentration of the reaction buffer HEPES in the final product (as an impurity), the Sep-Pak Light C18 gives a separation factor of approximately 1:2000 (factor of 0.0005). The DMSO content in the final product vial amounts to 0.055 mg (110 mg DMSO × 0.0005), which is below the PDE limit for a Class 3 solvent.
