# Supplementary material for: Automated Synthesis of [68Ga]Ga-FAPI-46 on a Scintomics GRP Synthesizer
Source: Pharmaceuticals (Basel). 2023 Aug 11;16(8):1138. doi: 10.3390/ph16081138 (PMC10459240; doi:10.3390/ph16081138)
Supplement: Supplementary file 1 [file pharmaceuticals-16-01138-s001.zip › pharmaceuticals-2529005-supplementary.pdf]

Stability Study of [^{68}Ga]Ga-FAPI-46 Batch 1

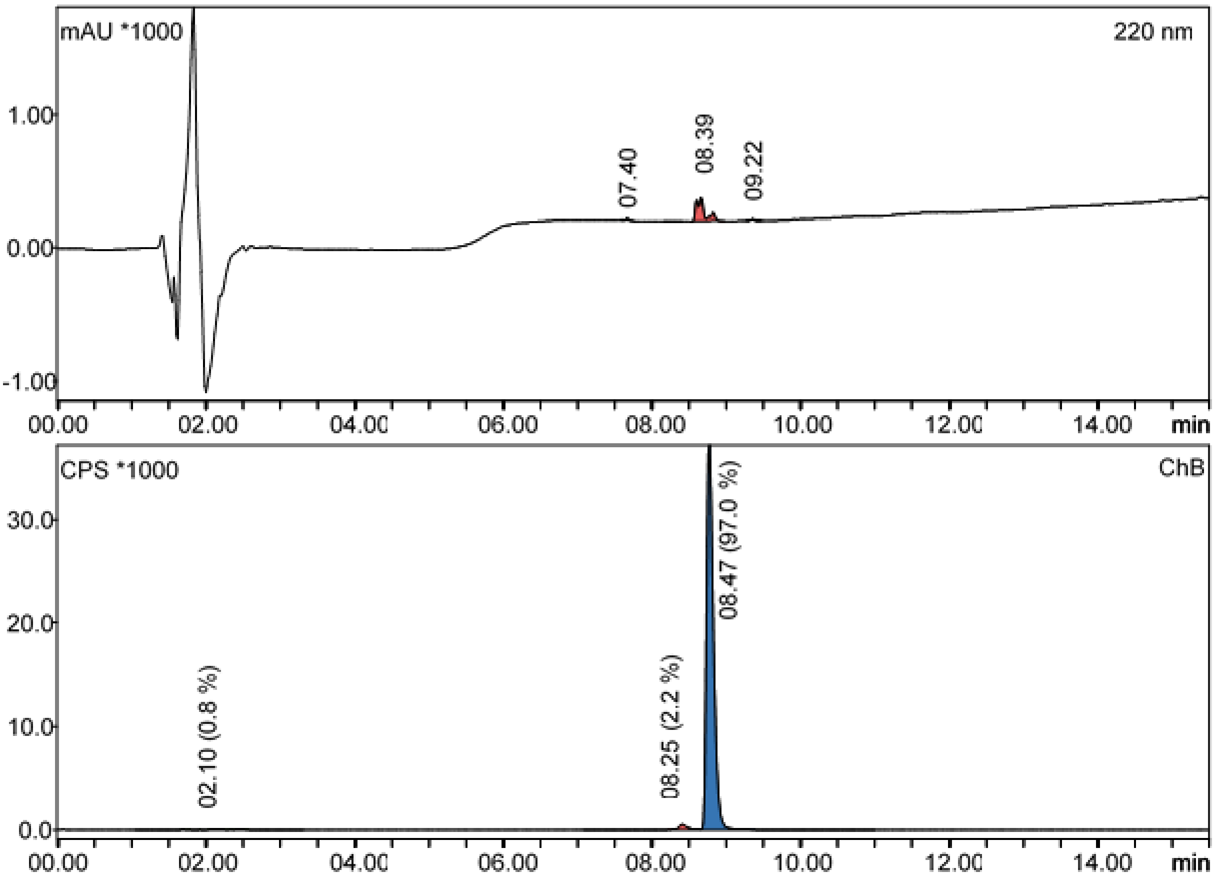

Figure S1. *Stability study batch 1: Radio-HPLC chromatogram of the [^{68}Ga]Ga-FAPI-46 product solution after EOS. UV trace (λ= 220 nm): peak at 7.40 min: 60.4 mAU*s; peak at 8.39: 1446,9 mAU*s; peak at 9.22 min: 36.2 mAU*s.*

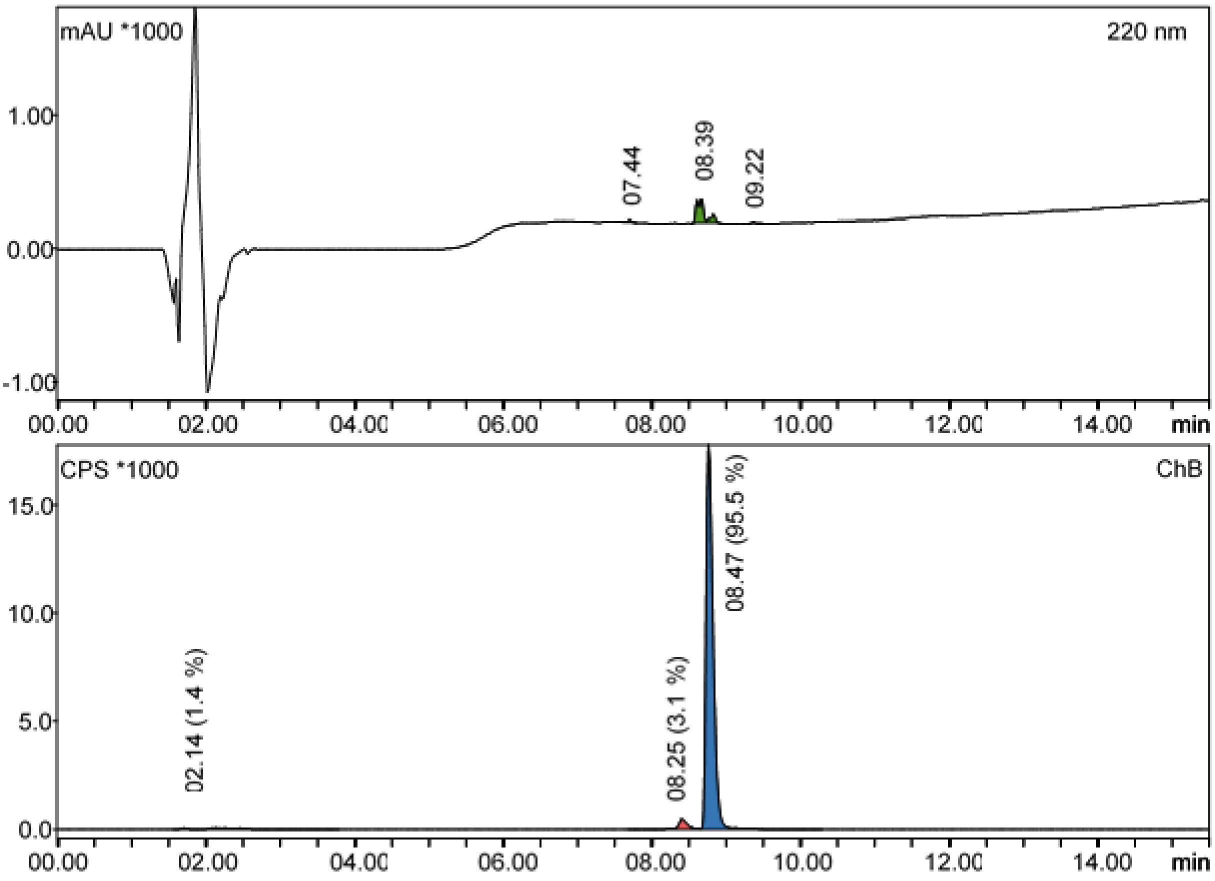

Figure S2. *Stability study batch 1. Radio-HPLC chromatogram of the [^{68}Ga]Ga-FAPI-46 product solution 1 h after EOS. UV trace (λ= 220 nm): peak at 7.44 min: 69.0 mAU*s; peak at 8.39: 1477.0 mAU*s; peak at 9.22 min: 38.2 mAU*s.*

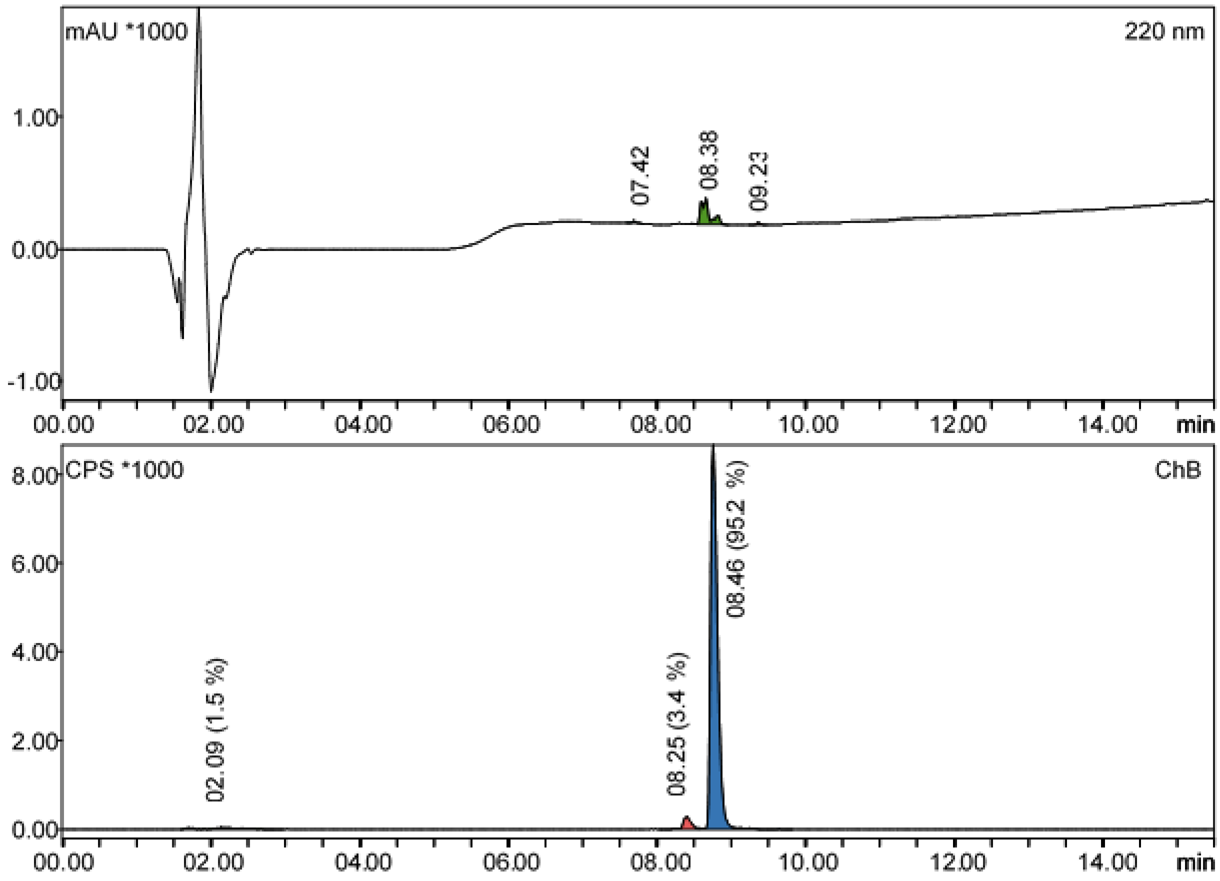

Figure S3. *Stability study batch 1. Radio-HPLC chromatogram of the [^{68}Ga]Ga-FAPI-46 product solution 2 hours after EOS. UV trace (λ= 220 nm): peak at 7.42 min: 65.9 mAU*s; peak at 8.38: 1491,5 mAU*s; peak at 9.23 min: 40.3 mAU*s.*

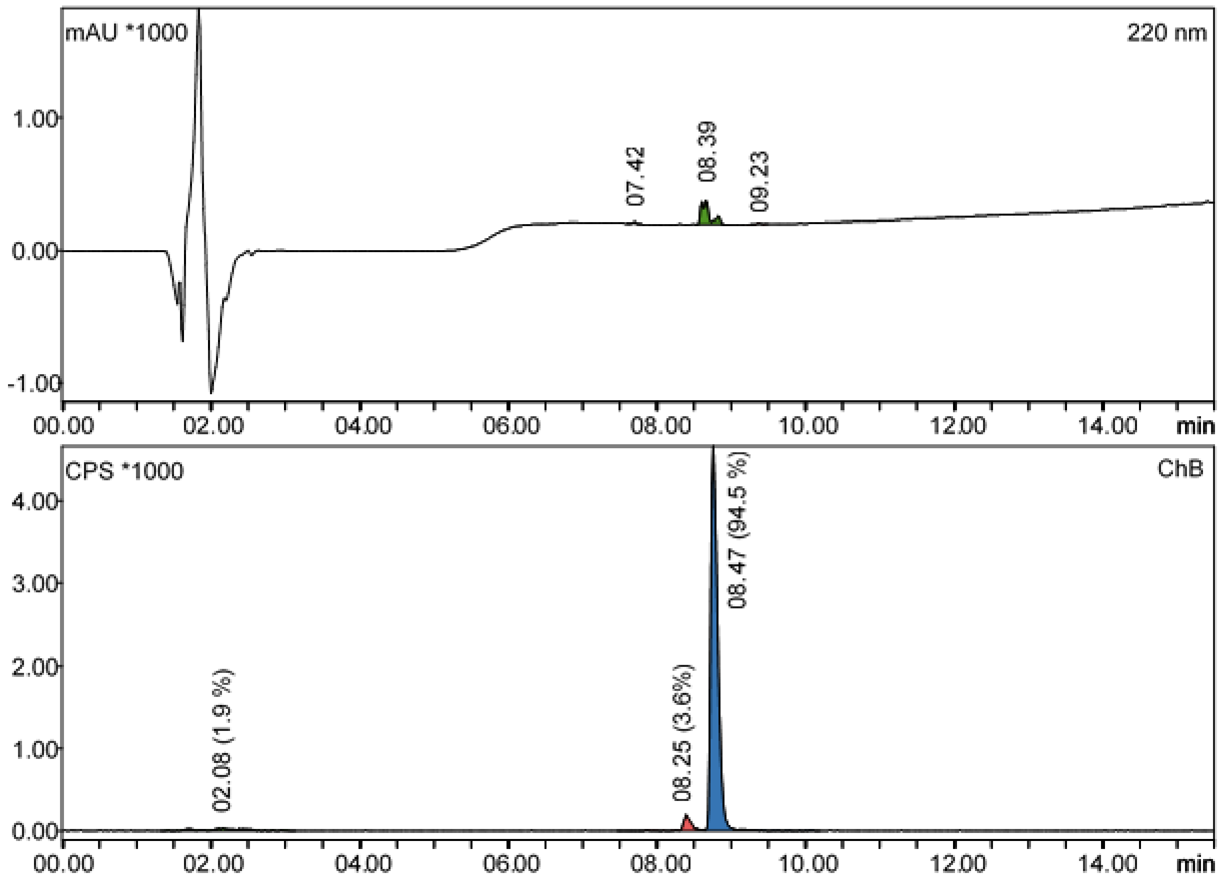

Figure S4. *Stability study batch 1. Radio-HPLC chromatogram of the [^{68}Ga]Ga-FAPI-46 product solution 3 hours after EOS. UV trace (λ= 220 nm): peak at 7.42 min: 59.5 mAU*s; peak at 8.39: 1450.4 mAU*s; peak at 9.23 min: 39.3 mAU*s.*

Stability Study of [^{68}Ga]Ga-FAPI-46 Batch 2:

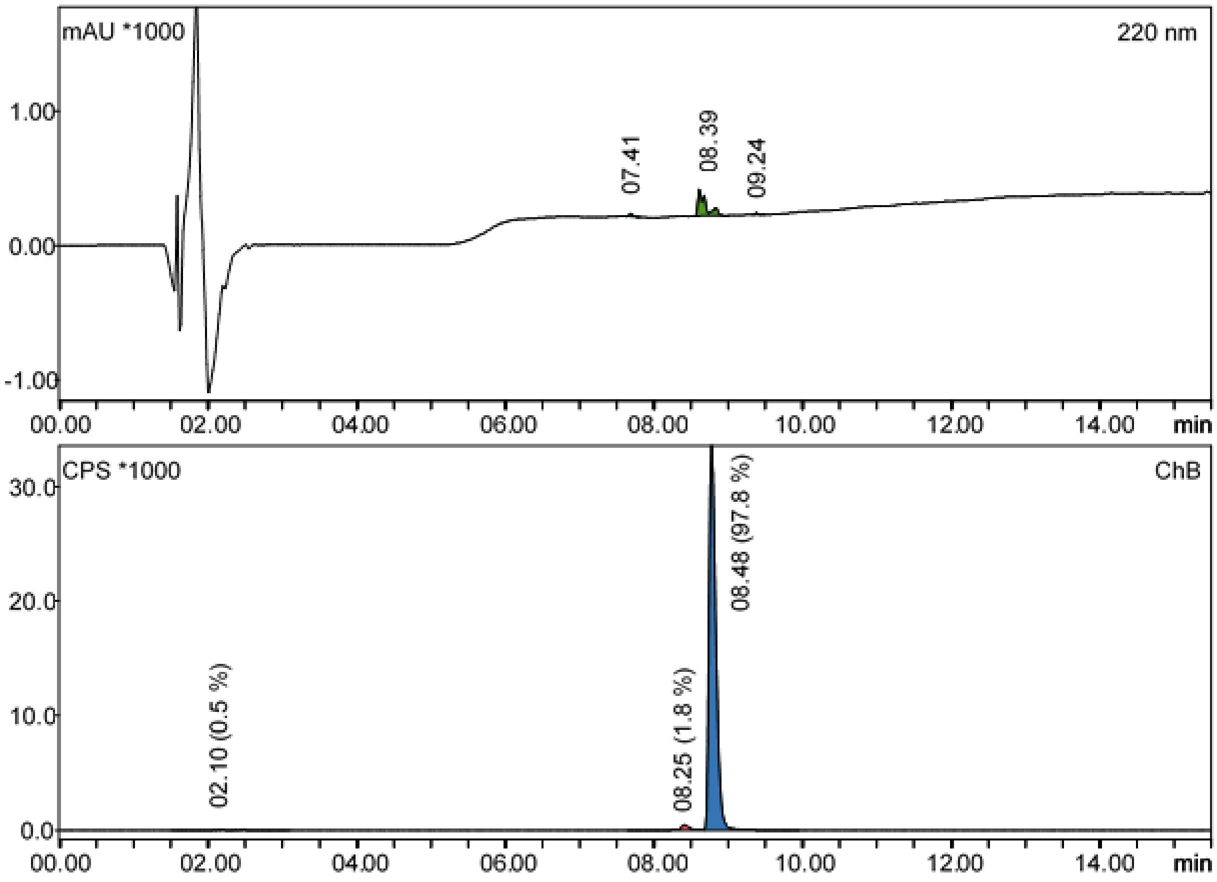

Figure S5. *Stability study batch 2. Radio-HPLC chromatogram of the [^{68}Ga]Ga-FAPI-46 product solution after EOS. UV trace (λ= 220 nm): peak at 7.41 min: 62.7 mAU*s; peak at 8.39: 1386.3 mAU*s; peak at 9.24 min: 35.7 mAU*s.*

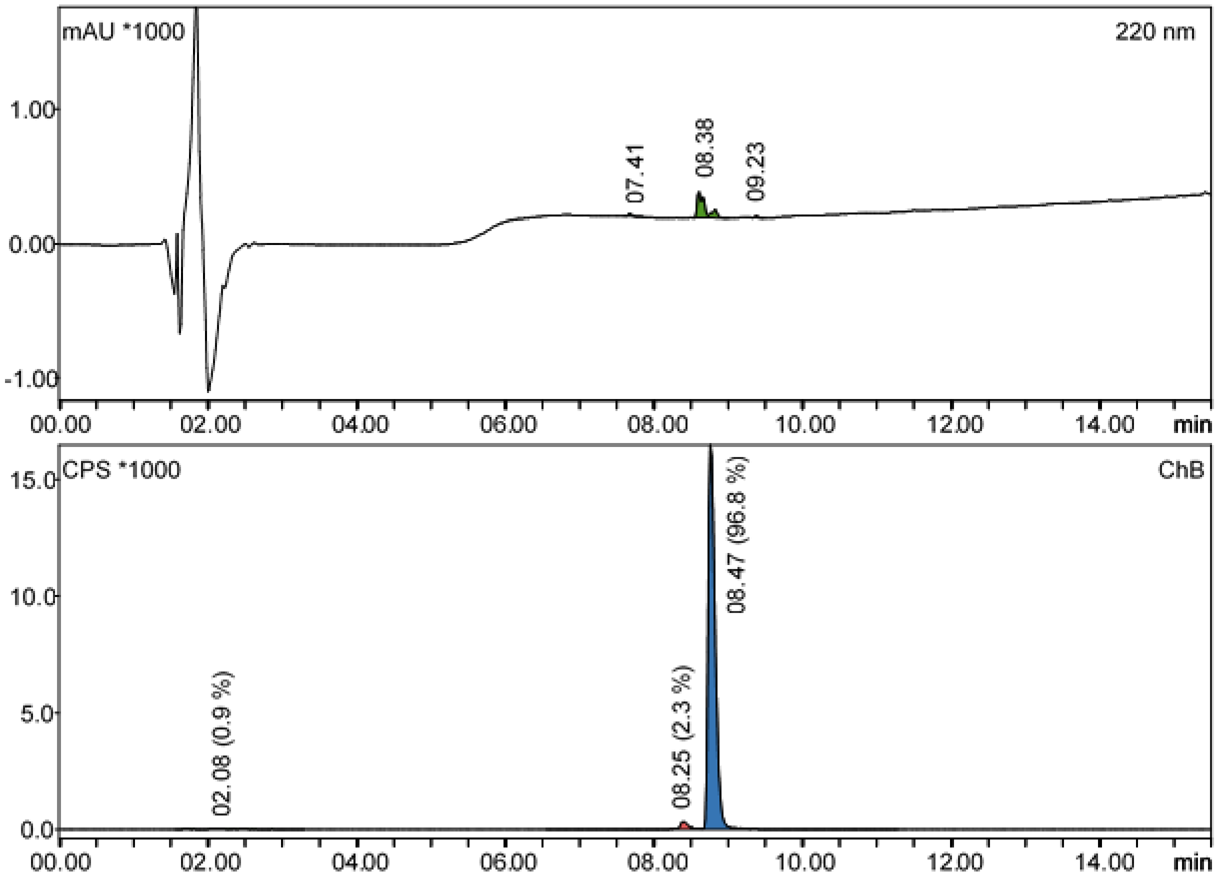

Figure S6. *Stability study batch 2. Radio-HPLC chromatogram of the [^{68}Ga]Ga-FAPI-46 product solution 1 h after EOS. UV trace (λ= 220 nm): peak at 7.41 min: 71.4 mAU*s; peak at 8.38: 1382.8 mAU*s; peak at 9.23 min: 38.1 mAU*s.*

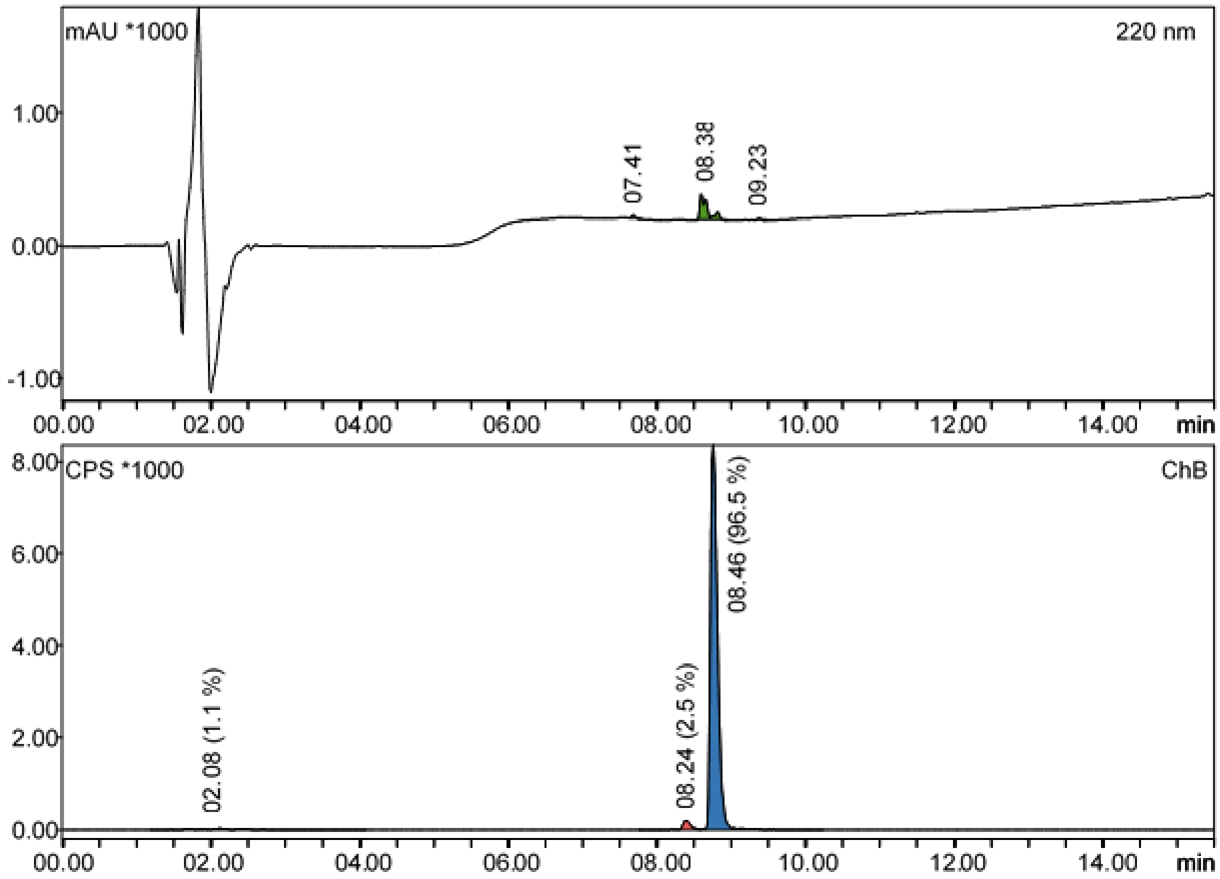

Figure S7. *Stability study batch 2. Radio-HPLC chromatogram of the [^{68}Ga]Ga-FAPI-46 product solution 2 h after EOS. UV trace (λ= 220 nm): peak at 7.41 min: 65.6 mAU*s; peak at 8.38: 1371.5 mAU*s; peak at 9.23 min: 36.2 mAU*s.*

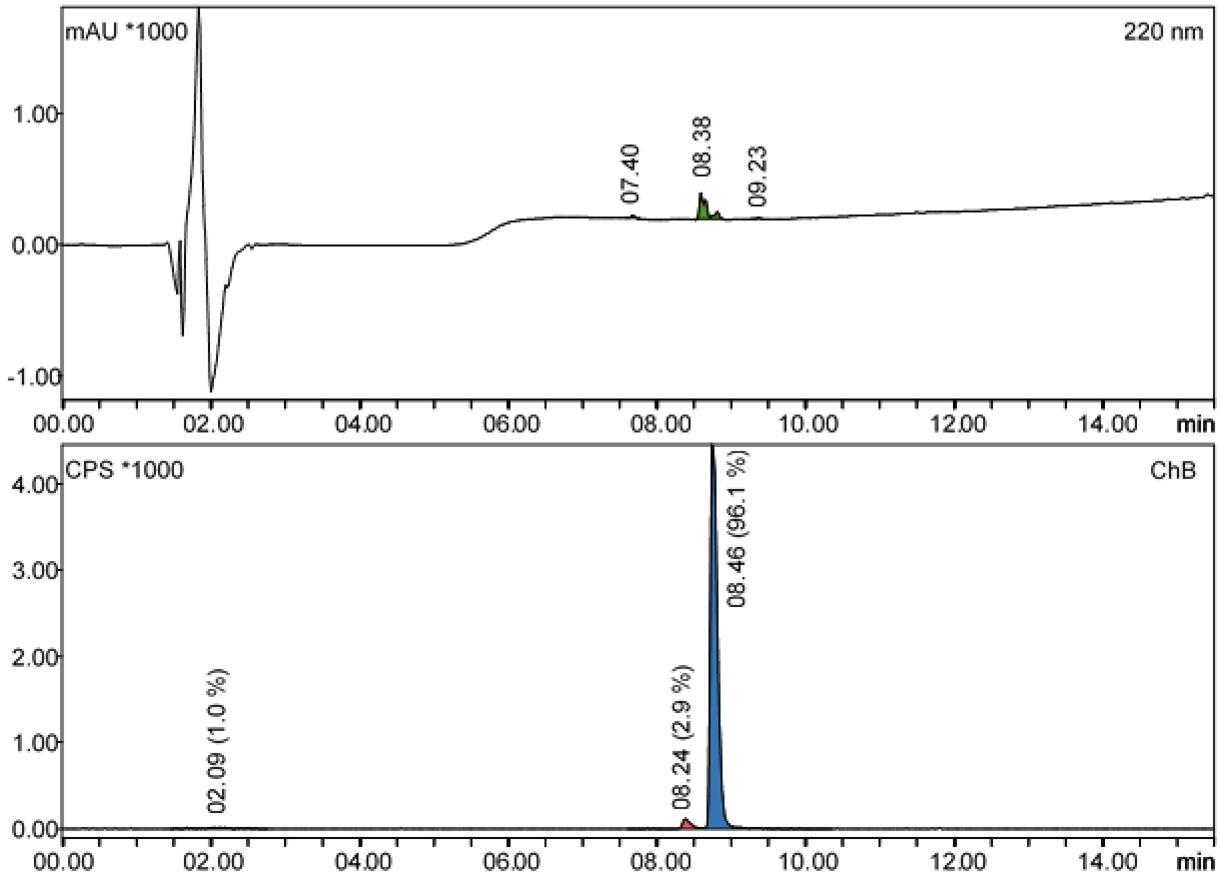

Figure S8. *Stability study batch 2. Radio-HPLC chromatogram of the [^{68}Ga]Ga-FAPI-46 product solution 3 h after EOS. UV trace (λ= 220 nm): peak at 7.40 min: 69.3 mAU*s; peak at 8.38: 1344.1 mAU*s; peak at 9.23 min: 38.3 mAU*s.*

Stability Study of [^{68}Ga]Ga-FAPI-46 Batch 3:

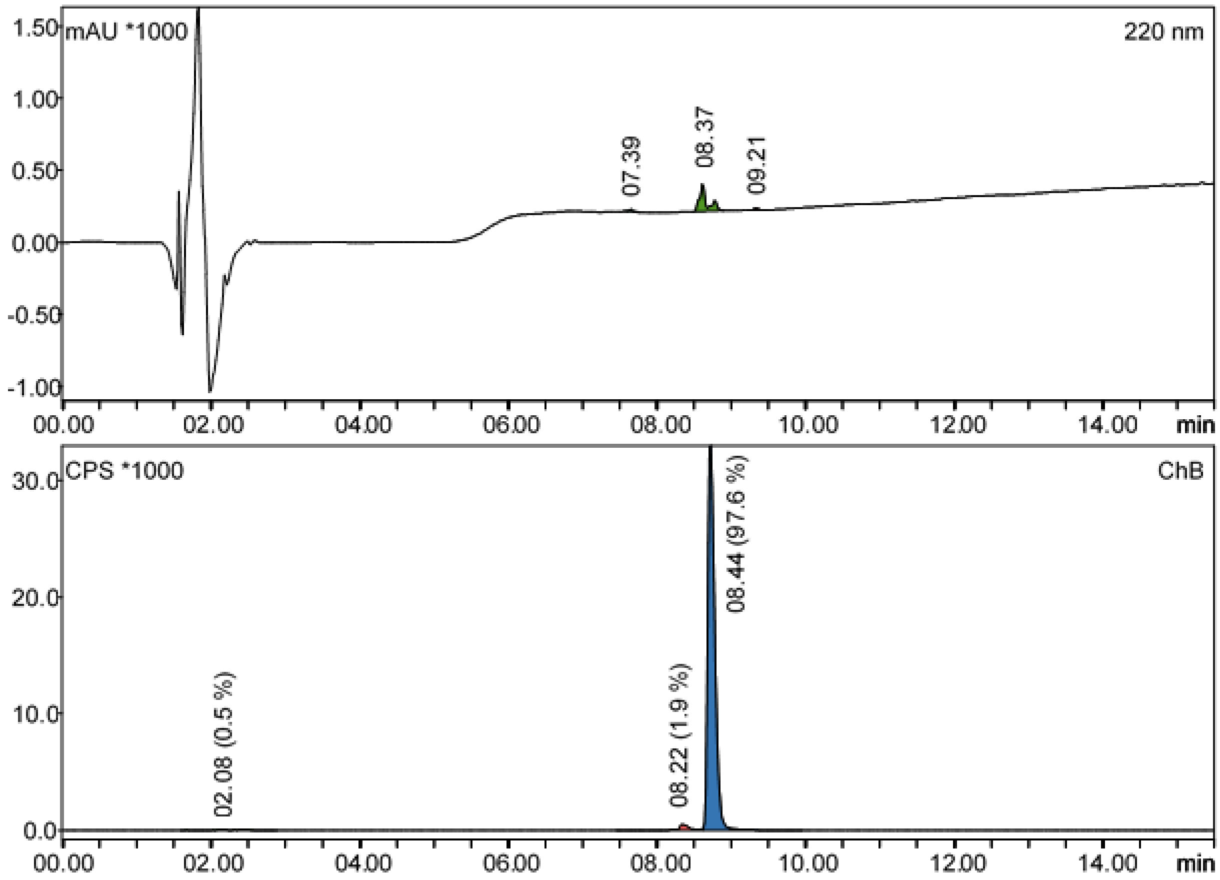

Figure S9. *Stability study batch 3. Radio-HPLC chromatogram of the [^{68}Ga]Ga-FAPI-46 product solution after EOS. UV trace (λ= 220 nm): peak at 7.39 min: 56.1 mAU*s; peak at 8.37: 1262.8 mAU*s; peak at 9.21 min: 36.3 mAU*s.*

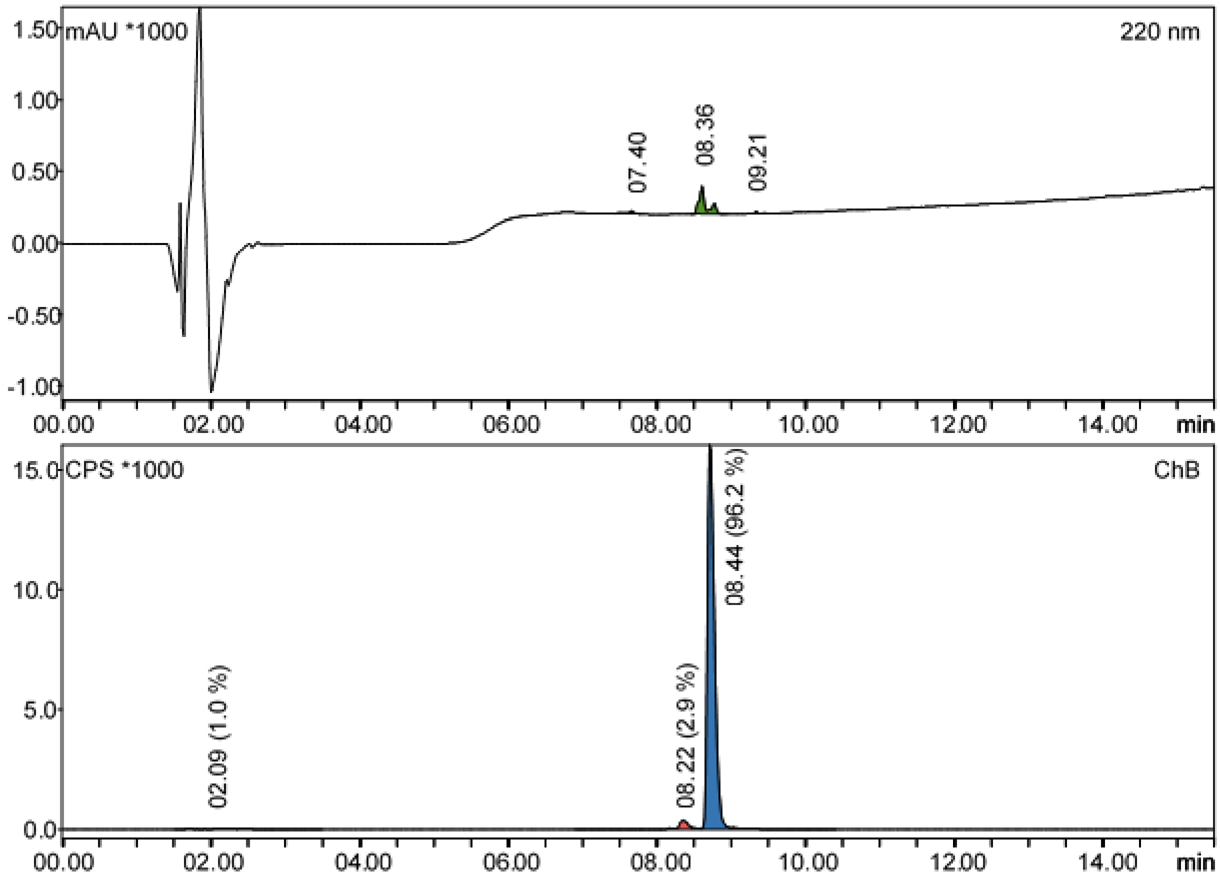

Figure S10. *Stability study batch 3. Radio-HPLC chromatogram of the [^{68}Ga]Ga-FAPI-46 product solution 1 h after EOS. UV trace (λ= 220 nm): peak at 7.40 min: 57.6 mAU*s; peak at 8.36: 1245.7 mAU*s; peak at 9.21 min: 36.9 mAU*s.*

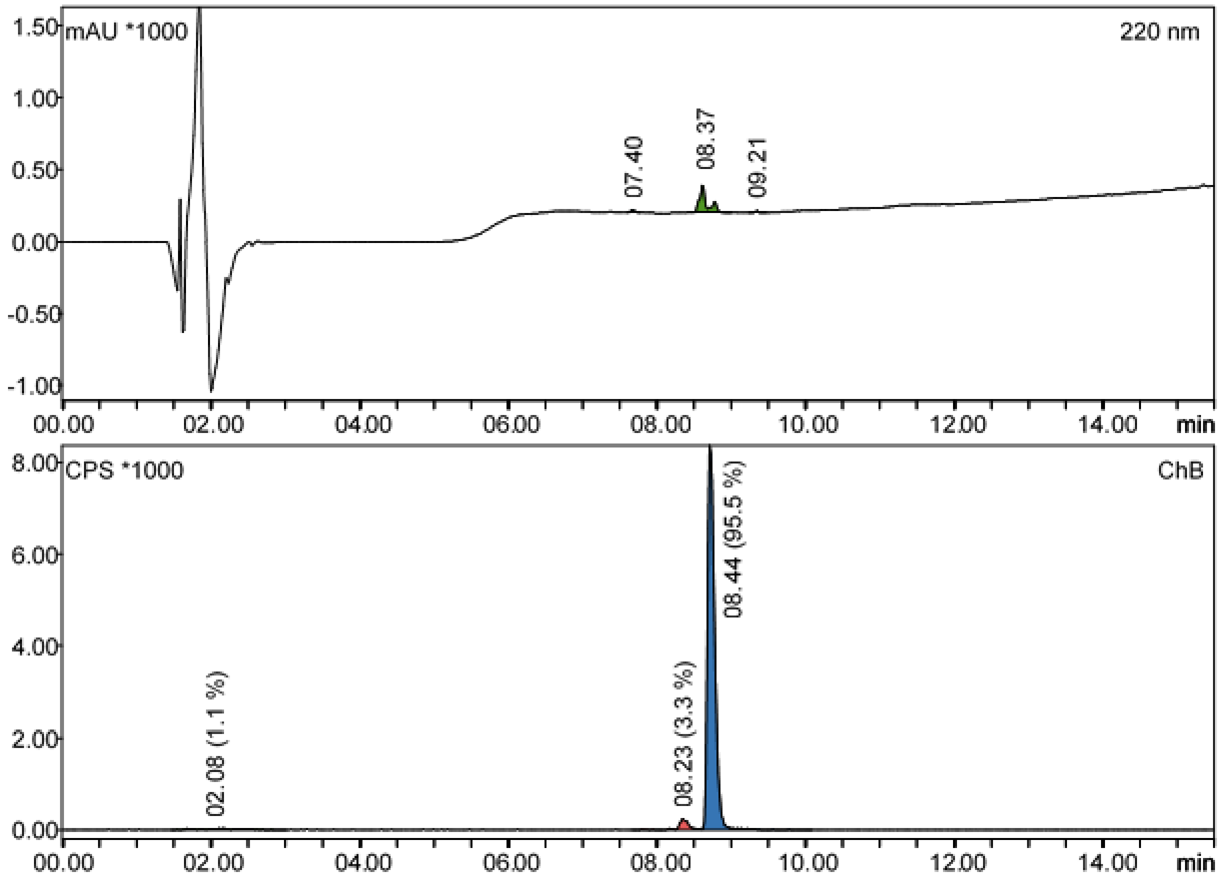

Figure S11. *Stability study batch 3. Radio-HPLC chromatogram of the [^{68}Ga]Ga-FAPI-46 product solution 2 h after EOS. UV trace (λ= 220 nm): peak at 7.40 min: 56.9 mAU*s; peak at 8.37: 1255.4 mAU*s; peak at 9.21 min: 34.3 mAU*s.*

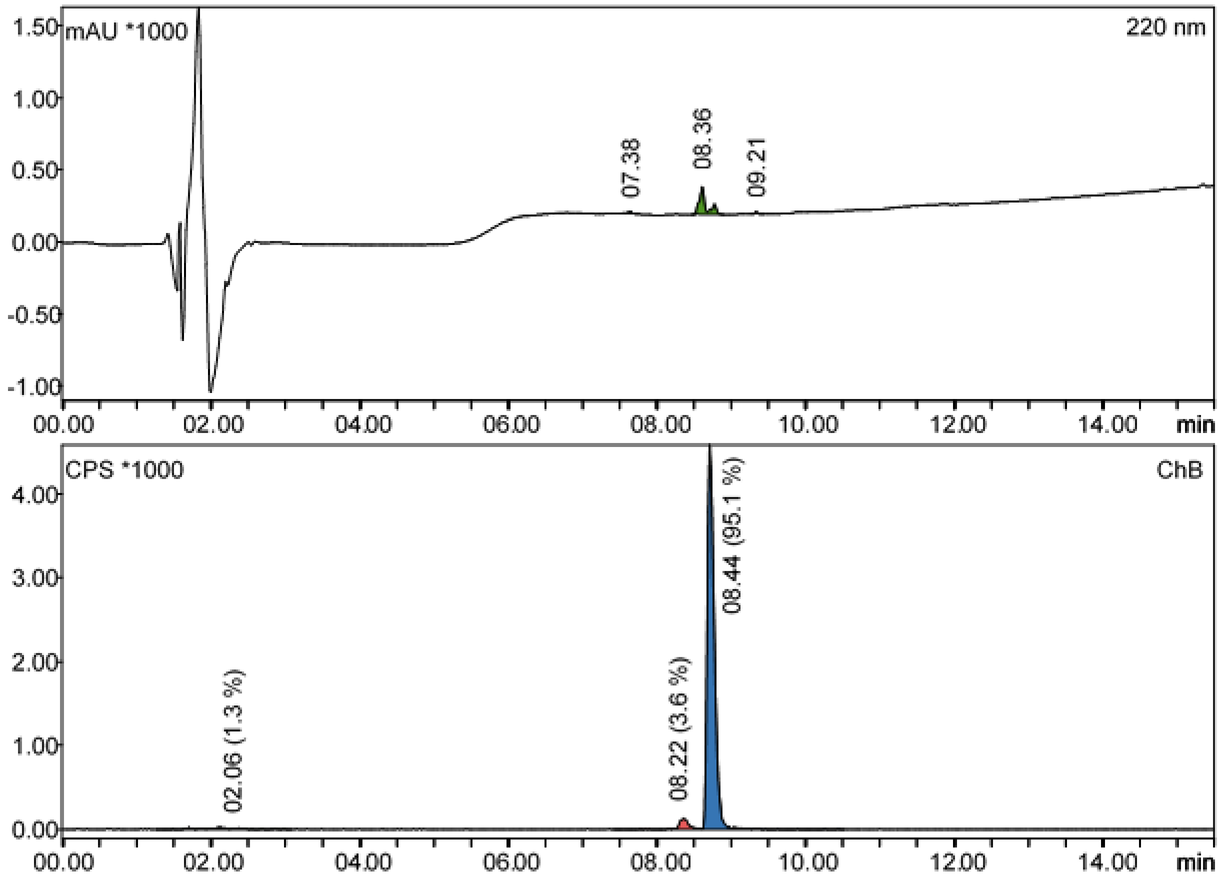

Figure S12. *Stability study batch 3. Radio-HPLC chromatogram of the [^{68}Ga]Ga-FAPI-46 product solution 3 h after EOS. UV trace (λ= 220 nm): peak at 7.38 min: 51.6 mAU*s; peak at 8.36: 1227.6 mAU*s; peak at 9.21 min: 39.1 mAU*s.*
